# Acidic Extracellular pH Promotes Activation of Integrin α_v_β_3_

**DOI:** 10.1371/journal.pone.0015746

**Published:** 2011-01-19

**Authors:** Ranjani K. Paradise, Douglas A. Lauffenburger, Krystyn J. Van Vliet

**Affiliations:** 1 Department of Biological Engineering, Massachusetts Institute of Technology, Cambridge, Massachusetts, United States of America; 2 Department of Materials Science and Engineering, Massachusetts Institute of Technology, Cambridge, Massachusetts, United States of America; University Paris Sud, France

## Abstract

Acidic extracellular pH is characteristic of the cell microenvironment in several important physiological and pathological contexts. Although it is well established that acidic extracellular pH can have profound effects on processes such as cell adhesion and migration, the underlying molecular mechanisms are largely unknown. Integrin receptors physically connect cells to the extracellular matrix, and are thus likely to modulate cell responses to extracellular conditions. Here, we examine the role of acidic extracellular pH in regulating activation of integrin α_v_β_3_. Through computational molecular dynamics simulations, we find that acidic extracellular pH promotes opening of the α_v_β_3_ headpiece, indicating that acidic pH can thereby facilitate integrin activation. This prediction is consistent with our flow cytometry and atomic force microscope-mediated force spectroscopy assays of integrin α_v_β_3_ on live cells, which both demonstrate that acidic pH promotes activation at the intact cell surface. Finally, quantification of cell morphology and migration measurements shows that acidic extracellular pH affects cell behavior in a manner that is consistent with increased integrin activation. Taken together, these computational and experimental results suggest a new and complementary mechanism of integrin activation regulation, with associated implications for cell adhesion and migration in regions of altered pH that are relevant to wound healing and cancer.

## Introduction

Binding between cells and the extracellular matrix (ECM) is critical to complex processes such as cell adhesion and migration. This binding is mediated by interactions between cell surface integrin receptors and ECM ligands. Integrins are heterodimeric α/β transmembrane receptors which bind to ECM ligands such as fibronectin [1]. These receptors contain several metal ion binding sites; three of these sites, termed LIMBS, MIDAS, and ADMIDAS, are involved in regulation of integrin-ligand binding [2], [3]. Intracellularly, integrins can link to the actin cytoskeleton via a multi-protein assembly and also interact with signaling proteins that regulate processes such as cell survival and proliferation [4].

**Figure 1:**
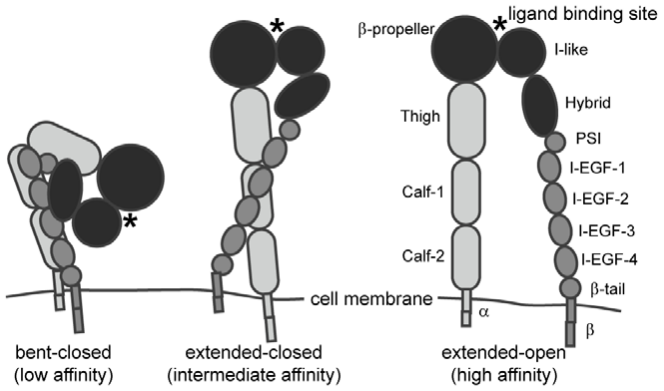
Integrins exhibit three distinct conformations correlated with binding affinity. Headpiece domains are depicted in black. In the low affinity conformation, the integrin leg domains are bent and the headpiece is closed. In the intermediate affinity conformation, the leg domains are extended and the headpiece is closed. In the high affinity conformation the leg domains are extended, and the headpiece is open.

Integrins undergo large-scale conformational changes in order to attain a high-affinity configuration during the process of integrin activation. These receptors are currently understood to exist in equilibrium among three main conformational states (Fig. 1). In the low-affinity state, the extracellular leg domains are bent and the headpiece is closed, with an acute angle between the I-like and hybrid domains. This conformation generally exhibits little to no binding to biological ligands [5], [6], [7], [8], but can bind to small RGD peptides in solution [9]. In the high-affinity conformation, the leg domains are extended and separated, and the headpiece is open. The third conformation, with extended legs and a closed headpiece, is expected to be of intermediate affinity [5].

Integrin activation can be regulated by signals from the extracellular (“outside-in”) and intracellular (“inside-out”) environments. During outside-in activation, the headpiece-tailpiece interface is destabilized by headpiece opening. This is followed by extension and separation of the leg domains, resulting in adoption of the extended-open conformation [5], [7]. Outside-in activation can be regulated by divalent cations; for example, Mn^2+^ promotes activation while Ca^2+^ stabilizes the low-affinity conformation [5], [10], [11]. Integrins can also be activated by ligand or antibody binding [5], [12]. Inside-out activation begins with separation of the α and β tails and results in adoption of the extended-closed conformation [5], and is regulated by intracellular signals such as talin binding to the integrin cytoplasmic domain [6], [8].

It is well known that extracellular pH can become acidic in several biological contexts. For example, while normal physiological pH is 7.4, the average extracellular pH in the tumor environment is generally in the range of 6.2–6.9 [13], [14], [15], [16]. In early stages of wound healing, the extracellular pH is in the range of 5.7–6.1 [17]. In addition, a cell can locally acidify its environment through the action of the Na^+^/H^+^ ion exchanger NHE1, which extrudes an intracellular H^+^ ion in exchange for an extracellular Na^+^ ion. Interestingly, it has been shown that NHE1 localizes to adhesion sites [18], [19], and could thus selectively acidify the extracellular environment proximal to the integrin receptors. In motile cells, NHE1 localizes to leading edge membrane protrusions [20], thereby creating a pH gradient at the single cell level, with a lower pH at the leading edge [21], [22]. It should be noted that NHE1 can also regulate cell migration via mechanisms independent of its function as an ion exchanger [18], although its role as a proton pump is most relevant to this study.

Acidic extracellular pH can affect several cell processes, including adhesion and migration. For example, adhesion between neutrophils and endothelial cells was found to strengthen when the extracellular environment was acidified [23]. In addition, human melanoma cells exhibited more lamellipodia and stronger adhesion at lower extracellular pH, while migration speed was maximum at intermediate pH [24]. Human melanoma cells also displayed enhanced invasiveness and increased secretion of proteases and proangiogenic factors in response to acidic extracellular pH [25]. Furthermore, mouse metastatic melanoma cells increased in size and elongation ratio, and displayed increased migration capacity and gelatinase secretion after exposure to acidic extracellular pH [26]. In human breast cancer cells, the number and length of filopodia increased at acidic pH [27], and in mouse microglial cells, acidic pH was noted to promote cytoskeletal rearrangement and stress fiber formation [28]. Finally, cell adhesion and proliferation increased when fibroblasts, MG-63, or Saos-2 cells were cultured on vinyl phosphonic acid (VPA)/acrylamide gels with a higher proportion of acidic VPA [29].

While some of these observations have been attributed to changes in intracellular protein expression or protease secretion, the molecular mechanisms facilitating these changes are unknown. In particular, the role of integrin receptors in mediating the observed cellular responses to acidic pH has not been explored in depth. To our knowledge, to date only two studies have addressed this possibility. Stock et al. speculated that an extracellular acid-induced strengthening of the integrin-ligand bond could explain their observed effects of pH on melanoma cell adhesion and migration, but did not directly investigate this hypothesis [24]. Lehenkari and Horton observed that the rupture force between integrin α_v_β_3_ and a GRGDSP peptide increased at acidic pH, but did not assess the possible reasons that this occurs [30]. In addition, this result has not been repeated or supported by other molecular-level experiments. Here, we consider the hypothesis that acidic extracellular pH can directly alter integrin conformation, thereby affecting integrin-ligand binding. Specifically, we employ molecular dynamics simulations, and flow cytometry and molecular force spectroscopy experiments, to investigate the effect of acidic extracellular pH on activation of integrin α_v_β_3_. We also conduct cell-level morphology and migration experiments to assess the consequent effects on cell behavior.

## Results

### Acidic extracellular pH promotes integrin headpiece opening in molecular dynamics simulations

**Figure 2:**
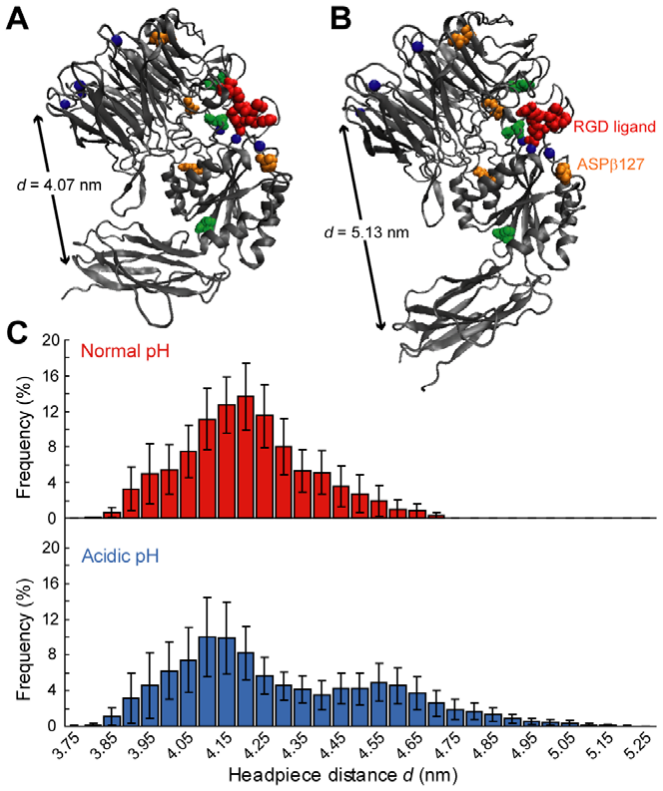
Acidic extracellular pH promotes α_v_β_3_ headpiece opening in molecular dynamics simulations. (A) Atomistic rendering of the α_v_β_3_ headpiece in a closed conformation. Spheres depict Mg^2+^ ions (blue), RGD ligand (red), residues with elevated pKa that were protonated at both normal and acidic pH (green) or protonated at acidic pH only (orange). Arrow indicates headpiece opening distance. (B) Atomistic rendering of the α_v_β_3_ headpiece in a partially open conformation. (C) Histograms of headpiece opening distances from all recorded frames of every simulation trajectory. Frequencies are displayed as the average of the eight simulations at each pH condition. Error bars represent standard error of the mean (SEM).

To gain atomistic understanding of how acidic extracellular pH affects integrin conformation, we employed molecular dynamics (MD) simulations. The crystal structure used for these simulations was the extracellular domain of integrin α_v_β_3_ in complex with an RGD peptide [9]. Only the integrin headpiece was simulated to reduce the solvated system to a computationally tractable size (Fig. 2A). In the α_v_β_3_ crystal structure, the headpiece is closed; this configuration provides an opportunity to study the effect of extracellular pH on headpiece opening *in silico*, independently of other effects that may be coupled *in vitro* and *in vivo*.

We used Multi-Conformation Continuum Electrostatics (MCCE) to predict pK_a_ values for all titratable residues in the α_v_β_3_ headpiece. MCCE predicts pK_a_ values for residues within the protein context, which can differ appreciably from pK_a_ of these residues in isolation [31], [32]. To simulate normal physiological pH, we protonated residues with pK_a_>8.4. For an effective acidic pH, we protonated residues with pK_a_>6.2. From the definition of pK_a_, these residues would be protonated at a pH below the threshold pKa of 6.2 for a majority of integrins (>50%), thus computationally approximating an acidic pH on that order; we refer to this state hereafter as “acidic pH.” MCCE results indicated seven amino acids with pK_a_ values that were elevated from their expected solution values, and were thus protonated in our simulations (Fig. 2A–B, Table 1).

**Table 1:** Residues with elevated pKa values as predicted by MCCE.

Residue	Predicted pKa
GLUα123	11.933
ASPβ217	8.53
HISβ244	8.492
HISα91	6.223
ASPα186	7.109
ASPβ127	6.692
HISβ274	7.103

GLUα123, ASPβ217, and HISβ244 were protonated both the normal and acidic pH systems. HISα91, ASPα186, ASPβ127, and HISβ274 were protonated in the acidic system only.

We conducted eight replicate MD simulations (8 ns duration each) of each pH system, and quantified the amount of headpiece opening that occurred. Puklin-Faucher et al. previously observed partial headpiece opening within 6–8 ns of MD simulations of α_v_β_3_ in complex with the fibronectin III_10_ domain [33]. Despite some differences in simulation details, replication of those simulations to the extent possible confirmed that the maximum headpiece opening observed in our simulations is comparable to that reported by Puklin-Faucher et al. At normal physiological pH, a histogram of headpiece opening distances *d* exhibits a single peak centered at *d* = 4.2 nm. At acidic pH, the histogram exhibits two peaks, centered at *d* = 4.1 nm and 4.55 nm (Fig. 2C). The emergence of this second peak demonstrates that the α_v_β_3_ headpiece more frequently samples a partially open state at acidic pH. Although the α_v_β_3_ headpiece did not reach the fully open conformation in the timescale of our simulations, the partial headpiece opening we observed is expected to be on the pathway to complete opening [33]. Therefore, these results indicate that acidic extracellular pH promotes opening of the α_v_β_3_ headpiece.

**Figure 3:**
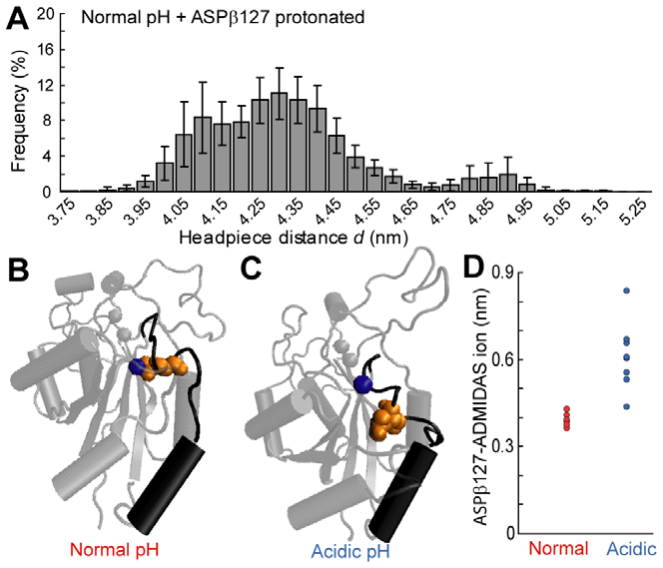
Increased headpiece opening at acidic extracellular pH can be attributed in part to ASPβ127 protonation. (A) Histogram of headpiece opening distances displayed as in Fig. 2. (B) Snapshot of the α_v_β_3_ I-like domain in the normal pH system demonstrating ASPβ127-ADMIDAS ion coordination. ADMIDAS ion is displayed as a blue sphere, ASPβ127 is displayed in orange spheres, and the α1-β1 loop and α1 helix are displayed in black. (C) Snapshot of the α_v_β_3_ I-like domain in the acidic pH system demonstrating the lack of ASPβ127-ADMIDAS ion coordination. (D) Average distances between the centers-of-mass of the ADMIDAS ion and ASPβ127 in each simulation trajectory. Error bars (SEM) are within data points.

ASPβ127, which is protonated in our acidic pH system, is located in the α1-β1 loop at the top of the α1 helix in the I-like domain, and can coordinate the divalent cation at the ADMIDAS site. As movements within the α1 helix have been implicated in headpiece opening [11], we investigated the role of ASPβ127 in the acid-induced headpiece opening observed in our simulations. We established a simulation system identical to the normal physiological pH system, except for the additional protonation of ASPβ127. A histogram of headpiece opening distances for this system revealed three peaks, two of which were shifted to higher distances than the opening distance observed at normal pH (Fig. 3A). This indicates that the headpiece opening observed at acidic pH could be at least partially attributed to protonation of the ASPβ127 residue.

At normal physiological pH, ASPβ127 is negatively charged and coordinates the ADMIDAS ion (Fig. 3B). When ASPβ127 is protonated at acidic pH, it has neutral charge and no longer coordinates the ADMIDAS ion (Fig. 3C). This is demonstrated by the distance between ASPβ127 and the ADMIDAS ion, which is much shorter at normal pH (Fig. 3D). We suggest that the loss of ASPβ127-ADMIDAS ion coordination at acidic pH increases the likelihood that the α1-β1 loop and α1 helix sample displacements that promote headpiece opening.

Our simulations demonstrate that acidic pH promotes opening of the α_v_β_3_ headpiece, attributable in part to protonation of ASPβ127 in the I-like domain. Headpiece opening in the bent-closed conformation destabilizes the bent state and is expected to lead to integrin extension [7], [34], [35]. Therefore, our MD simulations indicate that acidic pH will shift the integrin conformational equilibrium towards the fully activated extended-open state, independently of other possible downstream effects of extracellular acidification.

### The level of activated α_v_β_3_ on live cell surfaces increases after exposure to acidic pH

**Figure 4:**
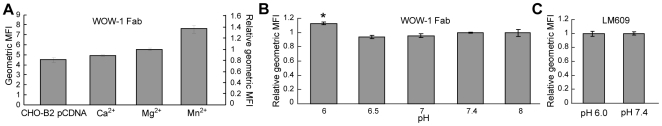
Flow cytometry measurements demonstrate increased level of activated α_v_β_3_ on live cell surfaces after exposure to acidic extracellular pH. (A) WOW-1 Fab binding in the presence of various divalent cations. Data are displayed as the average geometric MFI of triplicate samples from a single representative experiment (primary axis), as well as the normalized geometric MFI (see Methods) expressed relative to the average value measured in the presence of Mg^2+^ ions (secondary axis), to enable direct comparison to (B). (B) WOW-1 Fab binding after exposure to pH 6.0–8.0. Data are normalized geometric MFI (see Methods) at each pH expressed relative to the average value measured at pH 7.4. Averaged data displayed here were calculated from at least two independent experiments at each pH. Asterisk indicates p<0.01 with respect to all other pH conditions. (C) LM609 binding after exposure to pH 6.0 or 7.4. Data is expressed as in (B). All error bars represent SEM.

To investigate the effect of acidic pH on integrin activation on live cell surfaces, we conducted flow cytometry experiments with the antibody WOW-1 Fab, which preferentially binds to activated α_v_β_3_ [36]. We measured the level of activated α_v_β_3_ on live α_v_β_3_ CHO-B2 cell surfaces after incubation in buffer pH over the range 6.0–8.0. Cell shrinkage and detachment indicated a decrease in cell viability at pH≤5.5; therefore these lower pH values were not assessed via flow cytometry. Control experiments with various divalent cations confirmed WOW-1 specificity as a marker of activated α_v_β_3_ (Fig. 4A). Ca^2+^ ions stabilize the inactive conformation [5], [10], [11], and cells in the presence of Ca^2+^ displayed low levels of WOW-1 binding. Mn^2+^ ions promote activation [5], [10], [11], and cells exposed to Mn^2+^ displayed the highest level of WOW-1 binding. An intermediate level of WOW-1 binding was observed in the presence of Mg^2+^, and all pH experiments were performed with this ion. As expected, WOW-1 binding was lowest on CHO-B2 pCDNA cells, which do not express the integrin β_3_ subunit. Although the difference in fluorescence intensity between Mn^2+^-stimulated cells and negative control CHO-B2 pCDNA cells was small (see Fig. S1 for histograms), the fold change we observed is comparable to that reported by others for this antibody [8], [36].

The levels of WOW-1 binding after exposure to pH 6.5, 7.0, and 8.0 differed insignificantly from that at physiological pH 7.4; however, exposure to pH 6.0 resulted in a significantly higher level of binding (p<0.01, Fig. 4B). We used antibody LM609, which binds both activated and inactivated α_v_β_3_, to assess whether exposure to pH 6.0 changes the total cell surface expression of α_v_β_3_. LM609 binding after exposure to pH 6.0 was indistinguishable from that at pH 7.4 (Fig. 4C), confirming no measurable change in the overall level of integrin expression at acidic pH. Therefore, our flow cytometry results indicate that exposure to an acidic pH of 6.0 results in an increased level of activated α_v_β_3_ receptors on live cell surfaces.

### Frequency of α_v_β_3_-RGD binding increases at acidic pH, but rupture forces are unaffected

To further quantify the conformational state of α_v_β_3_ on single cell surfaces, we also conducted atomic force microscope (AFM)-mediated molecular force spectroscopy; this molecular force spectroscopy is defined here as measurement of the distribution of unbinding forces *F*_R_ between ligand-receptor complexes upon application of load at a given applied loading rate. Previous studies have utilized AFM force spectroscopy to investigate α_4_β_1_-VCAM-1 and α_5_β_1_-fibronectin interactions on U937 and K562 cells, respectively. In those reports, the applied force required to rupture the induced ligand-receptor complex *F*_R_ and binding frequency *f*_b_ increased after integrin activation [37], [38]. Therefore, we measured both rupture forces and binding frequencies of α_v_β_3_-RGD on live α_v_β_3_ CHO-B2 cells, as a function of extracellular pH.

**Figure 5:**
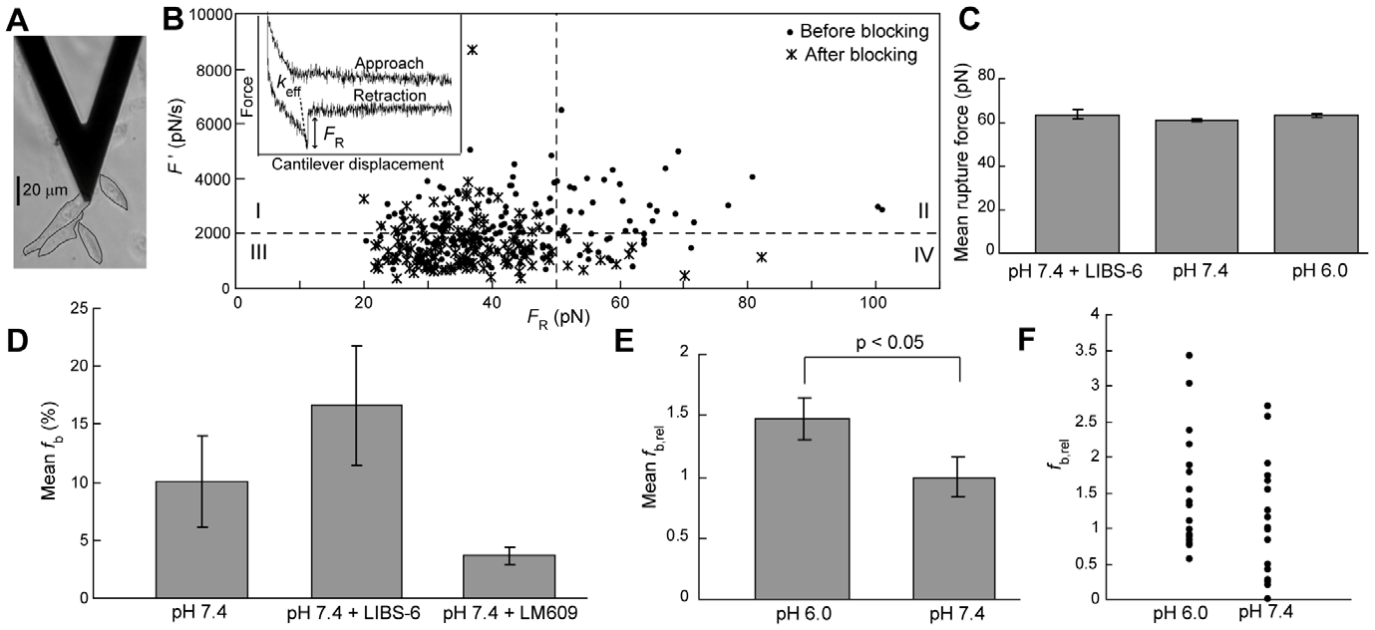
AFM-mediated molecular force spectroscopy measurements demonstrate that acidic extracellular pH increases frequency of α_v_β_3_-RGD binding on live cell surfaces, but does not alter rupture forces. (A) Optical image of an AFM cantilever positioned over an α_v_β_3_ CHO-B2 cell. (B) Rupture forces *F*_R_ and effective loading rates *F′* for all unbinding events measured before and after LM609 blocking in a single experiment. Inset depicts a representative force-displacement curve. (C) Mean rupture forces measured at pH 7.4, pH 7.4 after α_v_β_3_ activation by LIBS-6, and pH 6.0. (D) Mean specific binding frequencies *f*_b_ at pH 7.4 before and after α_v_β_3_ activation by LIBS-6 or α_v_β_3_ blocking by LM609. (E) Mean relative specific binding frequencies *f*_b,rel_ at pH 6.0 and pH 7.4. (F) Single cell relative binding frequencies *f*_b,rel_ (one point per cell) used to calculate the average in (E). All error bars represent SEM.

In our experiments, the RGD-functionalized AFM cantilever was positioned over live α_v_β_3_ CHO-B2 cells, far from each cell nucleus (Fig. 5A), and 200 force cycles were performed on each cell in a grid pattern. A representative force-displacement trace for a single force cycle is shown in the inset of Fig. 5B.

To distinguish specific α_v_β_3_-RGD interactions from nonspecific binding interactions, we conducted measurements on cells before and after incubation with the antibody LM609, which blocks the RGD binding site on α_v_β_3_. It is expected that specific α_v_β_3_-RGD interactions will exhibit rupture forces distributed around a characteristic value corresponding to the strength of this molecular complex at the particular loading rate used here [37], [38], [39]. The effective loading rate *F′* should also be distributed around a characteristic value that reflects the stretching behavior of the ligand-receptor complex, molecular linkers, and cell immediately before rupture. These characteristic distributions provided the opportunity to effectively gate the specific interactions for each cell, despite the inherently large variation in experimental responses.

Analysis of rupture forces and effective loading rates before and after blocking demonstrated that unbinding events with *F*_R_>50 pN and *F′*>2000 pN/s occurred frequently before, but not after, blocking the RGD binding site (Fig. 5B, quadrant II, 16.80±2.58% vs. only 0.49±0.49% (mean ± SEM)). Therefore, rupture events with these characteristics were assumed to be specific α_v_β_3_-RGD interactions, and events in quadrants I, III, and IV were assumed to be nonspecific (or, more accurately, to be events which could not be objectively identified as specific). Additional negative controls were performed with cantilevers functionalized with linker molecules, but without GRGDSPC. In identical experiments conducted with these cantilevers, only 3.68±1.75% (mean ± SEM) of the rupture events fell within quadrant II, thereby confirming that events with *F*_R_>50 pN and *F′*>2000 pN/s were specific interactions that occurred infrequently in negative control experiments. Only these specific interactions were included in further analysis.

We conducted AFM force spectroscopy experiments on cells at pH 7.4 and pH 6.0. We also conducted experiments at pH 7.4 after activating α_v_β_3_ with antibody LIBS-6 [12]. Analysis of specific rupture forces revealed no significant differences in mean *F*_R_ among these three conditions (Fig. 5C). The similarity in mean *F*_R_ with and without LIBS-6 indicates that in our system, α_v_β_3_-RGD rupture forces do not detectably increase when the integrin is activated.

We also measured the α_v_β_3_-RGD specific binding frequency *f*_b_. Mean *f*_b_ at pH 7.4 increased after incubation with LIBS-6, demonstrating that a shift towards the activated state in the α_v_β_3_ conformational equilibrium can be detected on intact cell surfaces as an increase in specific binding frequency. This is due to the much higher likelihood of the extended integrin conformations, as compared to the bent conformations, to bind the adhesive ligands. Mean *f*_b_ was very low after blocking with LM609, confirming the specificity of the interactions chosen for analysis (Fig. 5D).

We then normalized single-cell *f*_b_ values to the mean *f*_b_ measured at pH 7.4, to obtain the relative specific binding frequency *f*_b,rel_; this normalization enabled us to combine data from replicate experiments conducted with different cantilevers. Comparison of *f*_b,rel_ at pH 6.0 and pH 7.4 revealed that the mean *f*_b,rel_ was significantly higher at pH 6.0 (p<0.05, Fig. 5E), although there was considerable variation in *f*_b,rel_ among different cells within a given pH (Fig. 5F). This trend was maintained in each of the three independent experiments we conducted. The shift in mean *f*_b,rel_ cannot be explained by a change in the cell surface expression level of α_v_β_3_, as demonstrated by our flow cytometry results with antibody LM609 (Fig. 4C). In combination with the observation that binding frequency increases after activation by LIBS-6, these experimental data indicate that acidic extracellular pH shifts the α_v_β_3_ conformational equilibrium towards the activated state.

### Acidic extracellular pH influences cell spreading, morphology, and migration speed

**Figure 6:**
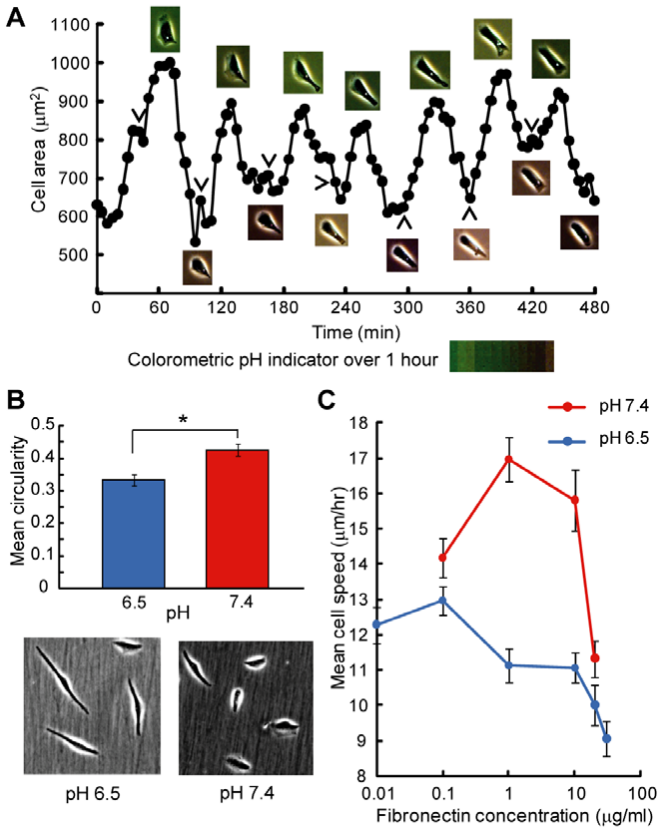
Cell spreading, morphology, and migration speed are altered at acidic extracellular pH. (A) Cell area for a representative cell over 8 hours, during which media pH was changed to pH 6.0 every hour. After each pH change, media pH drifted up to ∼7.2 due to presence of bicarbonate buffer, as evidenced by the color change of the pH indicator in the media (shown for 1 hour). Images are snapshots of the cell at area minima and maxima. Arrowheads indicate point immediately before media pH was brought down to pH 6.0. Each hour, cell area increased immediately after pH was dropped to 6.0, and decreased as pH drifted up. (B) Mean cell circularity 8 hours after changing media pH for adhered α_v_β_3_ CHO-B2 cells. Cell circularity was significantly lower at pH 6.5 than at pH 7.4 (asterisk indicates p = 0.0002). Images are representative for cells at pH 6.5 and pH 7.4. (C) Mean cell migration speed as a function of fibronectin coating concentration. Maximum migration speed occurred at a lower fibronectin concentration when cells were in acidic pH. Statistical significance of maximum migration speed value at pH 7.4: 0.1 vs. 1 µg/ml, p<0.05; 1 vs. 20 µg/ml, p<0.001; 1 vs. 30 µg/ml, p<0.001. For pH 6.5: 0.1 vs. 1 µg/ml, p<0.1; 0.1 vs. 10 µg/ml, p<0.05; 0.1 vs. 20 µg/ml, p<0.001; 0.1 vs. 30 µg/ml, p<0.001. All error bars represent SEM.

We next examined the effect of acidic extracellular pH on cell-level adhesion and migration. We first considered the dynamic cell response to pH changes by utilizing the media bicarbonate buffer to create a system in which pH varied over time. For these experiments, α_v_β_3_ CHO-B2 cells were seeded on fibronectin-coated glass-bottom dishes and allowed to adhere for 3 hours. Media pH was then changed to pH 6.0 every hour for 8 hours; between pH changes, the media pH increased from 6.0 up to ∼7.2 due to the bicarbonate buffer and the presence of 5% CO_2_. This pH increase was clearly visible as a change in color of the pH indicator present in the media (Fig. 6A colorbar). Each time the media was changed to pH 6.0, many cells responded within minutes by spreading and elongating. This caused the projected cell area to increase, reaching a maximum about 30 minutes after each induced acidification to pH 6.0 (Fig. 6A). As the media pH increased toward neutral pH, cells eventually began to shrink and thus the projected cell area decreased. This cell behavior was consistent and repeatable over 8 hours of media pH changes, resulting in oscillations of measured cell area that initiated within minutes. Consistent with these measurements of changes to cell morphology in the attached state, we also observed that the initial adhesion and spreading of cells seeded from the suspended state also varied with pH: cells exhibited a higher spread area 30 minutes after adhesion when seeded in acidic media, as compared to media at pH 7.4 (Fig. S2). To further explore cell spreading as a function of pH, we quantified membrane dynamics via kymography [40]. Analysis of kymographs showed that membrane protrusion velocity was decreased and protrusion lifetime was increased at acidic pH, as compared to pH 7.4 (Figure S3).

To measure the effects of acidic pH over longer timescales, we seeded α_v_β_3_ CHO-B2 cells on fibronectin-coated polystyrene dishes and allowed them to adhere for 3 hours before changing the media to pH 6.5 or 7.4. Media pH was then maintained over eight hours by using dishes with tight-fitting lids that eliminated air exchange and consequent bicarbonate buffer-mediated pH changes. Eight hours after the pH change, many cells at pH 6.5 had developed an elongated morphology (Fig. 6B images). This was quantified by calculating cell circularity, which ranges from 0–1. Circularity close to 1 indicates that the cell has a rounded morphology, while circularity close to 0 indicates an elongated or dendritic morphology. Consistent with observations from such images, the mean cell circularity was significantly lower at 6.5 than at pH 7.4 (Fig. 6B). When integrin α_v_β_3_ was blocked with soluble RGD peptides, mean cell circularity was high and the pH-dependent difference was eliminated. The same effect was observed for CHO-B2 pCDNA cells. When integrin α_v_β_3_ was activated with Mn^2+^ ions, mean cell circularity was low, and the pH-dependent difference was again eliminated (Fig. S4).

We also measured cell migration speed as a function of fibronectin coating concentration at pH 6.5 and pH 7.4. Cell migration speed is expected to be biphasic with respect to ligand density [41]; at low ligand densities, the cell is unable to gain sufficient traction for migration, while at higher than optimal ligand densities, migration is limited by the ability of the cell trailing edge to detach from the substratum [42]. Modulating cell-substratum adhesiveness by changing integrin expression level or activation state alters the ligand density required for maximum migration speed [43]. Our cell migration experiments were conducted for α_v_β_3_ CHO-B2 cells seeded on fibronectin-coated polystyrene dishes with tight-fitting lids to maintain media pH. For cells in media maintained at pH 7.4, peak migration speed occurred at 1 µg/ml fibronectin, compared to a peak migration speed at 0.1 µg/ml fibronectin for cells in pH 6.5 media (Fig. 6C). Although the maximum measured speed value was lower at pH 6.5 than at pH 7.4, this leftward shift of peak speed to lower fibronectin concentrations indicates that cells at acidic pH require less ligand to achieve an optimal balance between traction and detachment. We note that the ligand density required for peak migration speed was substrate dependent, and occurred at higher fibronectin concentrations for cells plated on fibronectin-functionalized glass (Fig. S5).

## Discussion

We have used MD simulations, flow cytometry experiments, AFM force spectroscopy, and cell-level morphology and migration experiments to assess the effect of acidic extracellular pH on integrin α_v_β_3_ conformation and α_v_β_3_ CHO-B2 cell behavior. MD simulations demonstrate that the α_v_β_3_ headpiece attains a partially open state more frequently at acidic pH than at normal physiological pH, possibly due to ASPβ127 protonation and the resulting loss of ASPβ127-ADMIDAS ion coordination (Figs. 2–3). Several previous experimental studies have been reported in which ASPβ127 in β_3_ or the equivalent residue in other β subunits was mutated to ALA, which results in loss of ADMIDAS ion coordination. Overall, previous results of this mutation with respect to different integrin conformations and binding capacities are inconsistent, varying with the beta subunit. Mutated α_2_β_1_ and α_5_β_1_ integrins are capable of binding, mainly exhibit a closed headpiece, and can be activated by antibodies [2], [44]. Mutated integrin α_L_β_2_ showed constitutive ligand binding, but remained in the bent conformation [45]. However, ASPβ127 mutation in α_IIb_β_3_ does not inhibit normal activation and binding [46]. Therefore, losing ASPβ127-ADMIDAS ion coordination in β_3_ does not necessarily impair integrin function. Furthermore, ALA substitution of the ASPβ127-equivalent residue in α_4_β_7_ resulted in constitutive activation [3]. This suggests that in some integrins, loss of coordination of the ASPβ127-equivalent residue to the ADMIDAS ion is sufficient to cause integrin activation. Protonation of this residue at acidic extracellular pH is one mechanism by which loss of ADMIDAS ion coordination can occur. Although ALA mutation studies complement our MD simulation findings, we note that protonated ASP can participate in side-chain hydrogen bonding, but ALA cannot.

Our flow cytometry results indicate an increased number of activated α_v_β_3_ integrins on live cell surfaces after exposure to pH 6.0 (Fig. 4). It is important to note that in our assay it was necessary to perform the antibody-labeling step at pH 7.4 to ensure maximal antibody binding. Our data at pH 6.0 indicate that the activated integrin conformation can persist to some extent after cells are returned to pH 7.4. This is consistent with the results of Tzima et al., which demonstrated that even after removal of an activating stimulus, α_v_β_3_ integrins stay activated long enough to be labeled with WOW-1 antibody fragment Fab (i.e., ∼30 min post-activating stimulus) [47]. However, the levels of activated α_v_β_3_ we measured after exposure to pH 6.5 and pH 7.0 were not higher than that at pH 7.4, indicating that some level of reversal in activation state does occur after cells are returned to pH 7.4. Accordingly, it is plausible that acid-induced integrin activation also initially occurred in the pH 6.5 and 7.0 conditions, though to not as great a magnitude as for pH 6.0, so that detection in this assay was obfuscated during the antibody binding step.

Our AFM force spectroscopy results further demonstrate that the specific binding frequency of α_v_β_3_ integrins on cell surfaces to the RGD ligand increases at acidic pH, indicating an increase in the number of activated integrins (Fig. 5). However, the mean rupture forces measured at pH 7.4 and pH 6.0 are very similar to that measured after α_v_β_3_ activation by LIBS-6. We can speculate on a number of reasons underlying our observation that mean *F*_R_ did not increase upon activation. First, integrins are conformationally dynamic, and rearrange to the high-affinity state upon ligand binding [5]. Therefore, if RGD bound to a bent-closed or extended-closed integrin, the receptor may have rearranged to the extended-open conformation during our experimental contact time (>100 ms). Second, the bent-closed (low-affinity) conformation displays very low binding in other assays [5], [6], [7], so this conformation might not have been sampled substantively in our experiments. Finally, if RGD bound to a bent-closed or extended-closed integrin, and the receptor did not change conformation before unbinding, it is probable that the resulting rupture forces would be less than 50 pN and would not be distinguishable from nonspecific interactions in our analysis. Therefore, although the RGD ligand theoretically could have bound to any of the three integrin conformations, the measured mean specific *F*_R_ would not necessarily reflect a shift in the conformational equilibrium. In contrast to our results, previous AFM force spectroscopy studies by Li et al. and Zhang et al. report that integrin activation increased the measured *F*_R_ for α_5_β_1_-fibronectin [38] and α_4_β_1_-VCAM-1 [37]. It is possible that there were fewer nonspecific interactions in those experiments, or that the rate of conformational switching is slower for the integrins they probed, enabling measurement of ligand unbinding from the low-affinity integrin conformation. We also note that Lehenkari and Horton reported an increase in α_v_β_3_-RGD *F*_R_ at acidic pH [30]. Although that early observation supports our conclusion that acidic pH promotes integrin activation, those results should be interpreted with caution. For example, in those experiments, the RGD peptides were noncovalently functionalized to the AFM cantilevers, which can result in detachment of RGD from the AFM probe during the repeated interactions with the cell surface and render it difficult to ensure specificity of measured rupture forces.

**Figure 7:**
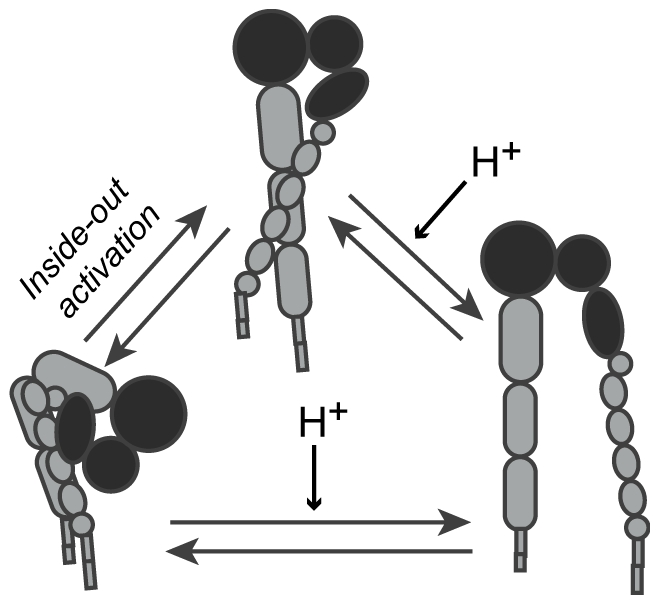
Model of pH-regulated integrin activation. Inside-out activation mechanisms can cause the bent-closed conformation to convert to the extended-closed conformation. Acidic extracellular pH can then promote headpiece opening to the extended-open conformation. In addition, acidic pH can stimulate headpiece opening on the bent-closed integrin, which is expected to lead to extension of the integrin legs. Through these dual mechanisms, acidic extracellular pH can shift the integrin conformational equilibrium to the high affinity extended-open state.

Our combined MD, flow cytometry, and AFM force spectroscopy data suggest a novel model by which acidic pH promotes headpiece opening of integrin α_v_β_3_ via protonation of ASPβ127. This could occur for integrins in the bent-closed or extended-closed conformation. The open headpiece destabilizes the bent conformation; therefore, acidic pH has the overall effect of shifting the integrin conformational equilibrium towards the high affinity extended-open state (Fig. 7). Conformational regulation by extracellular pH is a previously undescribed mechanism of integrin activation.

This model of pH-induced integrin activation is consistent with our cell-level measurements of cell area, circularity, and migration speed. Increased cell-substratum adhesiveness due to increased integrin activation should result in greater cell spreading, which is in agreement with our observation of oscillations in projected cell area in response to media pH changes (Fig. 6A). The dynamic, rapid, and reversible cell response to acidic pH indicates that this effect is not mediated by internal cell processes such as changes in protein expression, which would take longer to manifest, but is consistent with our biophysical model of pH-altered adhesiveness at the cell-substratum interface. Furthermore, the increase in cell area is not due to an increase in actin polymerization rate, as demonstrated by our kymography experiments that showed lamellipodial protrusion velocity to decrease in acidic pH (Fig. S3). In addition, protrusion lifetime increased in acidic pH, indicating enhanced lamellipodial stability, which is consistent with increased cell-substratum adhesiveness in this condition [40].

For our measurements of cell circularity and migration speed, the media was maintained at a steady acidic pH level over several hours. These experiments were conducted at pH 6.5 rather than pH 6.0. Although pH 6.0 is appropriate for experiments measuring molecular-level integrin activation and binding to antibodies or ligands, it may be too acidic for complex cell-level processes such as migration, which involve many subcellular systems that could be independently and deleteriously affected by acidic pH. Our MD data indicate that the critical amino acid ASPβ127 will be protonated on the majority of integrins as long as the extracellular pH is less than the predicted pKa of this residue within the integrin (6.692); therefore, we chose pH 6.5 for these long-timescale cell-level experiments.

Our observation that cells attained an elongated morphology characterized by lower circularity after long-timescale exposure to pH 6.5 (Fig. 6B) is consistent with Stock et al.'s observation of lower melanoma cell circularity in acidic pH [24] Further, we demonstrate that activation of integrin α_v_β_3_ with Mn^2+^ ions resulted in pH-independent low cell circularity (Fig. S4), which is consistent with Smith et al.'s observation that antibody-induced integrin activation caused T cells to adopt an elongated morphology [48]. These data indicate that the elongated morphology observed in our experiments can be attributed to increased integrin activation caused by acidic extracellular pH. We note that our model of acid-induced integrin activation is also consistent with Stock et al.'s speculation that acidic pH strengthened the integrin-ligand bond in their studies of melanoma cell morphology and migration [24]. Furthermore, our prediction that acidic pH could increase cell-substratum adhesiveness is consistent with several other reported effects of acidic pH on cell behavior, such as longer filopodia [27], increased adhesion efficiency [29], and increased cell spreading [26].

Our cell migration experiments demonstrated that the fibronectin concentration required for maximum migration speed is decreased at acidic pH (Fig. 6C). Palecek et al. showed that antibody-induced integrin activation in α_IIb_β_3_ CHO cells also decreased this critical ligand concentration to a similar extent as observed in our experiments [43]. Therefore, our measurement of a leftward shift of peak migration speed can be explained by a model of increased integrin activation at acidic pH, which results in increased cell-substratum adhesiveness and consequently less ligand required for efficient migration. However, in our experiments, we also observed that the maximum cell speed measured at pH 6.5 was lower than the maximum speed measured at pH 7.4. Such an effect was not observed in the studies by Palecek et al. [43], indicating that the reduction in peak migration speed we observed at acidic pH cannot be explained by increased integrin activation alone. Indeed, we speculate that this effect may be due in part to the decreased lamellipodial protrusion velocity we observed at acidic pH (Figure S3). Although this indicates that acidic pH may affect complex cell behaviors such as migration via more than one mechanism, it does not preclude the possibility of integrin activation via extracellular acidification. Finally, we note that it is unlikely that any of our cell-level results can be explained by acid-induced changes to fibronectin, as both guanidine hydrochloride-induced denaturation experiments and computational electrostatic calculations have demonstrated that the fibronectin protein structure is generally unchanged for pH 6.0 and higher [49], [50].

It is possible that our experimental changes to extracellular pH concurrently changed intracellular pH and associated cellular signaling events [51]. However, it is unlikely that our collective observations of increased integrin activation are due solely to alterations in intracellular signaling. First, our cell-free molecular dynamics simulations show that acidic extracellular pH causes opening of the integrin α_v_β_3_ headpiece in the absence of the transmembrane and intracellular domains, and in the absence of other intracellular proteins. Furthermore, our cell-level results are in agreement with those of Stock et al., who found that changes in intracellular pH could not explain their observed changes to cell morphology and migration [24]. While it remains possible that intracellular pH-induced changes to talin or other molecules may be a complementary mechanism of pH-induced integrin regulation, this does not exclude the possibility of direct extracellular pH-mediated integrin activation.

Acid-induced integrin activation poses interesting implications for cell adhesion and migration in several physiologically relevant contexts. In the tumor environment, the extracellular pH is generally acidic at the tumor periphery and is expected to increase in a gradient to physiological pH 7.4 away from the tumor site. Cells seeded on fibronectin gradients preferentially migrate towards areas of higher fibronectin concentration with concurrently higher overall adhesiveness [52]. Our prediction that regions of more acidic pH also confer higher effective adhesiveness suggests that the pH gradient in the tumor environment may alter migration dynamics of vascular endothelial cells or primary tumor cells in the near tumor site, providing an intriguing opportunity for further study. Further, the acidic pH in the early stages of wound healing could promote adhesion of inflammatory cells or directional migration of fibroblasts into the wound site. Finally, our results are also relevant to migration of normal healthy cells, even when bulk pH is ∼7.4. Due to the localization of the ion exchanger NHE1, the extracellular environment at the leading edge of polarized cells is more acidic than that at the trailing edge [21], [22]. Therefore, according to our proposed model, integrins will be activated in greater numbers at the leading edge, resulting in stronger adhesion. This will enhance the asymmetry in adhesiveness that is necessary for efficient migration [41], [53].

Integrin activation can be regulated by chemomechanical factors as diverse as divalent cation concentration [5], [10], [11], intracellular signaling [6], [8], and fluid shear stress [47]. Our computational and experimental findings demonstrate that integrin activation and binding affinity can also be modulated directly by the pH of the extracellular microenvironment. Together, these disparate activation mechanisms may enable integrins to finely tune binding affinity in response to intra- and extracellular cues, thereby facilitating precise regulation of cell adhesion and migration in varied environments. Elucidation of the ways in which various integrin activation mechanisms are coordinated will contribute to the challenging and exciting goal of understanding and modulating cell adhesion and migration in diverse physiological and pathological microenvironments.

## Materials and Methods

### Antibodies and reagents

Antibody LM609 was purchased from Millipore and Alexa 488 goat anti-mouse IgG (H+L) F(ab′)_2_ fragment was purchased from Invitrogen. WOW-1 Fab and LIBS-6 antibodies were provided by Dr. Sanford Shattil and Dr. Mark Ginsberg, respectively. SH-PEG-NH_2_ linker was purchased from Nanocs and Sulfo-LC-SPDP was purchased from Pierce Biotechnology. Dulbecco's Modified Eagle Medium (DMEM), antibiotics-antimyotics, non-essential amino acids, and zeocin were purchased from Invitrogen. Fibronectin from human plasma was purchased from Sigma. Fetal bovine serum was purchased from Hyclone.

### Cell culture

α_v_β_3_ CHO-B2 cells and CHO-B2 pCDNA cells were provided by Dr. Linda Griffith (Massachusetts Institute of Technology), as subcultures of cell lines developed by Dr. Jean Schwarzbauer (Princeton University) and Dr. Siobhan Corbett (University of Medicine and Dentistry of New Jersey), respectively. Cell culture media consisted of high-glucose bicarbonate-buffered DMEM containing L-glutamine and sodium pyruvate, supplemented with 10% fetal bovine serum, 1% antibiotics-antimyotics, and 1% non-essential amino acids. Media also included 500 µg/mL zeocin or 250 µg/mL G418 for α_v_β_3_ CHO-B2 or CHO-B2 pCDNA cells, respectively. Cells were maintained in an incubator at 37°C with 5% CO_2_.

### Molecular dynamics simulations

The input crystal structure for MD simulations was the extracellular portion of integrin α_v_β_3_ in complex with an RGD ligand (PDB ID 1L5G [9]). Mn^2+^ ions were replaced with Mg^2+^, and only the α propeller, β hybrid, and βA domains were simulated. Molecular dynamics simulations were conducted using GROMACS version 3.3 [54]. Simulations were performed under constant pressure and temperature, using periodic boundary conditions and Particle Mesh Ewald electrostatics with a short range interaction cutoff of 0.9 nm. The PRODRG server [55] was used to generate the RGD ligand topology. Multi-Conformation Continuum Electrostatics [31], [32] was used to predict pKa values for all the titratable amino acid residues in the integrin-ligand system. To simulate an effective acidic pH, all residues with pKa>6.2 were protonated. To simulate the normal physiological pH of ∼7.4, all residues with pKa>8.4 were protonated. GLUβ400 and GLUβ409 were not protonated in spite of their elevated pKa values; this is because these residues are close to the C-terminus of the simulation system and are thus artificially solvent exposed. A third simulation system designed to test the role of ASPβ127 had this residue protonated, in addition to all residues with pKa>8.4.

After protonation, the protein was solvated with Simple Point Charge water molecules in a box of dimensions 10.235 nm×11.513 nm×8.298 nm. Na^+^ and Cl^−^ were added at a physiological concentration of 0.137 M to provide charge neutrality. For each pH system, a two step steepest descents minimization of the X-ray diffraction structure was performed. In the first step, the integrin, RGD, and Mg^2+^ ions were held fixed and the maximum force in the system was reduced to less than 2000 kJ mol^−1^nm^−1^. In the second step, the full system was free to move and the maximum force in the system was reduced to less than 1500 kJ mol^−1^nm^−1^. After minimization, a 10 ps molecular dynamics simulation was performed with position restraints on the side chains of ARG_RGD_ and ASPα218, as suggested by Puklin-Faucher et al. [33]. Eight simulations of 10 ps duration were conducted for each pH, each with a different seed for random initialization of atomic velocities. The final frame at 10 ps was taken as input for further simulation, resulting in eight different input configurations for each pH. An MD simulation of 8 ns duration was performed for each input configuration. During MD simulations, ARG_RGD_ and ASPα218 position restrains were removed, and center of mass rotation and translation of the receptor were restrained.

To quantify the headpiece opening that occurred during MD simulations, we calculated the y-component of the distance *d* between a portion of the β-propeller domain (residues α250–438) and a portion of the hybrid domain (residues β55–106 and β356–434, Fig. 2A–B). The centers of mass of these regions were used for the distance calculation, and *d* was calculated at every recorded frame (every 5 ps) of each MD trajectory.

### Flow cytometry

Flow cytometry experiments were performed with α_v_β_3_ CHO-B2 cells, which have been engineered to express the integrin β_3_ subunit; the parental cell line CHO-B2 does not bind RGD [56]. CHO-B2 pCDNA cells were used as a negative control. WOW-1 Fab was used as a primary antibody to label activated α_v_β_3_, and LM609 was used to label all conformations of α_v_β_3_. Alexa 488 goat anti-mouse IgG (H+L) F(ab′)_2_ fragment was used as the secondary antibody.

The buffer used for all flow cytometry experiments consisted of 137 mM NaCl, 2.7 mM KCl, 3.3 mM NaH_2_PO_4_, 3.8 mM HEPES, 5.5 mM glucose, 1 mg/ml BSA, and 0.75 mM divalent cations (CaCl_2_, MgCl_2_, or MnCl_2_, as noted). Buffer was adjusted to desired pH using HCl and NaOH. Experiments were conducted at pH 6.0, 6.5, 7.0, 7.4, and 8.0. Cells were prepared for flow cytometry analysis as follows: cells were washed with (Ca^2+^, Mg^2+^-free) PBS, and detached with trypsin/EDTA. Trypsin was diluted, and cells were centrifuged at 800 rpm for 5 minutes. Cells were resuspended in buffer at desired pH, and 10 µl of each cell type was removed for counting in a hemocytometer. Cells were then centrifuged and resuspended at desired pH at a concentration of 2.3566×10^6^ cells/ml and incubated at room temperature for 20 minutes. All following steps were performed with pH 7.4 buffer. Cells were centrifuged and resuspended at 13.158×10^6^ cells/ml or 5×10^6^ cells/ml for WOW-1 Fab and LM609 experiments, respectively. 0.5×10^6^ cells were incubated with 12 µl WOW-1 Fab or 2 µg LM609 for 30 minutes at room temperature or on ice, respectively. During antibody incubations, cells were agitated every 10 minutes. After incubation, 200 µl buffer was added and cells were centrifuged. Cells were then resuspended in 100 µl buffer, and 100 µl of secondary antibody solution (1∶300 dilution in PBS) was added. Cells were incubated in secondary antibody for 30 minutes on ice, and agitated every 10 minutes. After incubation, 200 µl of buffer was added and cells were centrifuged. Cells were resuspended in 0.5 ml buffer and analyzed on a BD FACSCalibur flow cytometer. All experiments were performed with triplicate samples, and at least two independent experiments were performed for each pH (pH 6.0, N = 2; pH 6.5, N = 2; pH 7.0, N = 3; pH 7.4, N = 6; pH 8.0, N = 2). For each experiment, the average α_v_β_3_ CHO-B2 geometric mean fluorescence intensity (MFI) was normalized to the average CHO-B2 pCDNA geometric MFI. This quantity is referred to as the normalized geometric MFI.

### AFM force spectroscopy

All AFM force spectroscopy measurements were performed on an MFP-3D (Asylum Research, Inc.) system using Olympus TR400PB gold-coated silicon nitride cantilevers, with spring constant *k*∼25 pN/nm. Cantilever spring constant varied by approximately +/− 10% from this value. Cantilevers were cleaned in piranha solution (70% sulfuric acid, 30% hydrogen peroxide) and then rinsed thoroughly with 18 MΩ Millipore water. Rinses in following steps were performed in PBS+1 mM EDTA. To conjugate a polyethylene glycol (PEG) linker to the cantilever surface, a 1 mM solution of SH-PEG-NH_2_ (3.4 kDa) in PBS-EDTA was allowed to react with cantilevers for one hour at room temperature. Cantilevers were rinsed and then allowed to react with a solution of Sulfo-LC-SPDP cross-linker for 30 minutes at room temperature. Solution was prepared by dissolving Sulfo-LC-SPDP in ultrapure water at 20 mM, and diluting this solution 1∶40 in PBS-EDTA. Cantilevers were rinsed and then incubated in a 1 mg/ml solution of GRGDSPC peptide overnight at room temperature. Cantilevers were rinsed thoroughly once again to remove excess peptide before use. Functionalization was confirmed using fluorescent GRGDSPC peptides.

Experiments were performed at room temperature in buffer containing 137 mM NaCl, 2.7 mM Kcl, 3.3 mM NaH_2_PO_4_, 3.8 mM HEPES, and 1 mM MgCl_2_. Buffer was adjusted to pH 7.4 or pH 6.0 using HCl and NaOH. Experiments were conducted on α_v_β_3_ CHO-B2 cells adhered to 60 mm-diameter tissue-culture treated polystyrene Petri dishes (Falcon). Cells were incubated at room temperature for 20–30 minutes before measurements were taken. In experiments with LIBS-6 and LM609, antibody was added at 25 µg/ml or 20 µg/ml respectively, and allowed to react for 30 minutes at room temperature. For each cell, 200 force cycles were conducted in a 10×20 grid on a 2×2 µm area. For each force cycle, the AFM cantilever was positioned above a cell away from the nucleus. Cantilevers were moved toward the cell at a velocity *v* = 5 µm/s until a trigger force of 150 pN was reached. Cantilevers were then held on the cell surface for 0.1 s before retraction at 5 µm/s. All unbinding measurements were conducted at the same applied loading rate. Cantilevers were held above the cell surface for 1 s to allow cells to recover between measurements. Unbinding events were detected as jumps in the retraction portion of the force-displacement data.

Output force-displacement (*F-d*) data were analyzed with a customized Matlab script. For every *F-d* response exhibiting a visible unbinding event (jump in force during retraction), rupture force *F*_R_ was calculated as the difference between the average force following rupture and the minimum point at rupture. Effective loading rate *F′* was calculated as the product of the slope immediately before rupture (*k*_eff_) and the cantilever velocity *v* (Fig. 5B inset). Unbinding events with *F*_R_>50 pN and *F′*>2000 pN/s were taken to be specific α_v_β_3_-RGD interactions (Fig. 5B). Single-cell specific binding frequency *f_b_* was calculated as the number of specific unbinding events on a given cell normalized to the total number of unbinding events observed on that cell. Single-cell relative specific binding frequency *f_b,rel_* was calculated as the single-cell *f*_b_ normalized to the average *f*_b_ value at pH 7.4. Mean *f*_b_ and *f*_b,rel_ are averages taken over several cells at each condition. Three independent experiments comparing pH conditions were performed, with each replicate experiment at each pH including 200 spectra on each of five cells.

### Cell morphology and migration experiments

Unless otherwise noted, media for cell experiments consisted of high-glucose bicarbonate-buffered DMEM containing L-glutamine and sodium pyruvate, supplemented with 1% antibiotics-antimyotics, 1% non-essential amino acids, and 500 µg/mL zeocin. For migration experiments, 50 mm-diameter Petri dishes with tight-fitting lids (Pall Life Sciences) were coated with 0.1–30 µg/ml fibronectin in PBS for 1 hour at room temperature. Dishes were then rinsed twice with PBS. α_v_β_3_ CHO-B2 cells were plated on dishes in serum-free media at a density of approximately 6000 cells/cm^2^ and allowed to adhere for 3 hours before media was changed to pH 6.5 or 7.4. After media pH change, Petri dish lids were tightened to eliminate air exchange and consequent pH drift. Cells were imaged in phase contrast every 5 minutes for 8 hours in an incubator at 37°C. 10–15 unique fields were imaged for each experiment. Cell centroids were tracked using ImageJ, and cells that divided or touched other cells were excluded from analysis. Mean-squared displacements as a function of time (<*d^2^*(*t*)>) were calculated using the method of non-overlapping intervals [57], [58]. The root mean-squared displacement for the shortest interval was divided by the interval time (5 minutes) to obtain cell speed *S*. Mean-squared displacements as a function of time were fit to a persistent random walk model: <*d^2^*(*t*)> = 2*S* ^2^ *P*[*t*−*P*(1−*e* ^−*t*/*P*^)] [57]. Cells with a goodness-of-fit R^2^<0.5 were not included in the calculation of mean cell speed. At least 40 cells with R^2^>0.5 were analyzed for each condition. Cells plated on 10, 20, and 30 µg/ml fibronectin were also analyzed to measure circularity (4π*A*/*P* ^2^, where *A* is the projected cell area and *P* is the cell perimeter) at the 8-hour timepoint. Experiments at different fibronectin concentrations showed consistent results; presented data correspond to the fibronectin concentration of 10 µg/ml.

For pH oscillation experiment, 60 mm-diameter glass-bottom Petri dishes (MatTek) were coated with 30 µg/ml fibronectin as described above. α_v_β_3_ CHO-B2 cells were plated on dishes in serum-free media at a density of approximately 6000 cells/cm^2^ and allowed to adhere for 3 hours. After 3 hours, media was changed to pH 6.0 and refreshed to pH 6.0 every hour; between media changes, pH increased to ∼7.2. Cells were imaged in phase contrast every 5 minutes for 8 hours in an incubator at 37°C with 5% CO_2_. Projected cell area was measured using ImageJ. An independent experiment with 10 µg/ml fibronectin coating showed similar cell area oscillations (data not shown).

### Statistical Analysis

For comparison of two conditions, p values were calculated with an unpaired t-test. For comparison of three or more conditions, p values were calculated with a Bonferroni post-test following one-way ANOVA.

## Supporting Information

Figure S1 **Representative flow cytometry fluorescence intensity histograms illustrating WOW-1 Fab binding for CHO-B2 pCDNA cells, which do not express the integrin β_3_ subunit, and α_v_β_3_ CHO-B2 cells exposed to Mn^2+^, which activates integrin α_v_β_3_.**
(TIF)

Figure S2 **Mean spread area of α_v_β_3_ CHO-B2 cells plated from the suspended state into media at pH 6.0 or pH 7.4.** No. 1 glass coverslips or glass-bottom P60 dishes (MatTek) were coated with 15 µg/ml fibronectin in PBS for 1 hour at room temperature. Coverslips or dishes were then rinsed with PBS. α_v_β_3_ CHO-B2 cells were plated on coverslips or dishes in serum-free media with an initial pH of 6.0 or 7.4 and allowed to adhere for 30 minutes in an incubator at 37°C with 5% CO_2_ before imaging in phase contrast. Media initially set to pH 6.0 remained below pH 7.0 for the duration of the incubation. Cell area was measured using ImageJ. Two independent experiments were performed: in each experiment, approximately 40 cells were measured for each pH. Independent experiments showed consistent results, and data presented are from a single experiment. Results demonstrate that the mean cell area was significantly higher at pH 6.0 than at pH 7.4 (p = 0.0249). Error bars represent SEM.
(TIF)

Figure S3 **Kymography experiments demonstrate that membrane protrusion lifetime increases and protrusion velocity decreases at acidic extracellular pH.** (A) Example kymograph illustrating α_v_β_3_ CHO-B2 membrane dynamics. (B) Mean protrusion lifetime for cells in pH 6.5 or pH 7.4. (C) Mean protrusion velocity for cells in pH 6.5 or pH 7.4. Experiments were conducted as follows: glass-bottom P60 dishes (MatTek) were coated with 30 µg/ml fibronectin in PBS for 1 hour at room temperature. Dishes were then rinsed with PBS. α_v_β_3_ CHO-B2 cells were plated on coverslips or dishes in serum-free media and allowed to adhere for 3 hours before media was changed to bicarbonate-free serum-free media at pH 6.5 or 7.4. Cells were imaged in phase contrast at 40× magnification. Images were collected every 5 seconds for a duration of 25 minutes. Each kymograph was produced by drawing a one-pixel-wide line perpendicular to the cell membrane at an active lamellipod. The images along this line at all timepoints were then sequentially compiled into a single image, illustrating the membrane dynamics at that specific location on the cell. For each visible protrusion event on a kymograph, a straight line was drawn from the beginning of the event to its peak, or to the beginning of a plateau. Events with a height of less than 4 pixels were neglected. The slope of this line represents the protrusion velocity. Protrusion lifetime was quantified as the x-axis projection of this line, with the addition of plateau duration, if applicable. Two independent experiments were conducted: in each experiment, approximately 10–20 cells were imaged and 300–400 protrusion events were analyzed for each pH condition. Independent experiments showed consistent results, and data presented are from a single experiment. Results demonstrate that protrusion velocity is significantly decreased and protrusion lifetime is significantly increased at acidic pH (asterisks represent p<0.001). Error bars represent SEM.
(TIF)

Figure S4 **Mean cell circularity 8 hours after changing media pH for adhered α_v_β_3_ CHO-B2 cells or CHO-B2 pCDNA cells.** Petri dishes of 50 mm diameter with tight-fitting lids (Pall Life Sciences) were coated with 10 µg/ml fibronectin in PBS for 1 hour at room temperature. Dishes were then rinsed twice with PBS. α_v_β_3_ CHO-B2 or CHO-B2 pCDNA cells were plated on dishes in serum-free media at a density of approximately 6000 cells/cm^2^ and allowed to adhere for 2 hours before media was changed to bicarbonate-free serum-free media at pH 6.5 or 7.4. MnCl_2_ (1 mM) or soluble GRGDSPC peptide (200 µg/ml) was also added within this media exchange for some sample conditions, as indicated. Eight hours after the media change, optical images of cells were acquired and analyzed to measure circularity (4π*A*/*P* ^2^, where *A* is the projected cell area and *P* is the cell perimeter). At least 50 cells were analyzed for each condition. Results demonstrate that circularity is significantly decreased for α_v_β_3_ CHO-B2 cells in pH 6.5 (Columns 1 and 2, asterisk represents p<0.0001). There was no significant difference in circularity for α_v_β_3_ CHO-B2 cells in the presence of RGD at pH 6.5 vs. pH 7.4 (Columns 3 and 4), CHO-B2 pCDNA cells at pH 6.5 vs. pH 7.4 (Columns 5 and 6), or α_v_β_3_ CHO-B2 cells in the presence of Mn^2+^ at pH 6.5 vs. pH 7.4 (Columns 7 and 8).
(TIF)

Figure S5 **Mean migration speed as a function of fibronectin coating concentration on glass-bottom dishes for cells in media at pH 7.4.** Maximum migration speed occurred at 30 µg/ml fibronectin, compared to 1 µg/ml fibronectin when cells are plated on fibronectin-coated polystyrene (Figure 6C, main text). Statistical significance of maximum migration speed value: 10 vs. 30 µg/ml, p<0.01; 30 vs. 50 µg/ml, p<0.001; 30 vs. 60 µg/ml, p<0.001. Error bars represent SEM.
(TIF)
