# Supplementary figures and images for: Acidic Extracellular pH Promotes Activation of Integrin αvβ3
Source: PLoS One. 2011 Jan 19;6(1):e15746. doi: 10.1371/journal.pone.0015746 (PMC3023767; doi:10.1371/journal.pone.0015746)

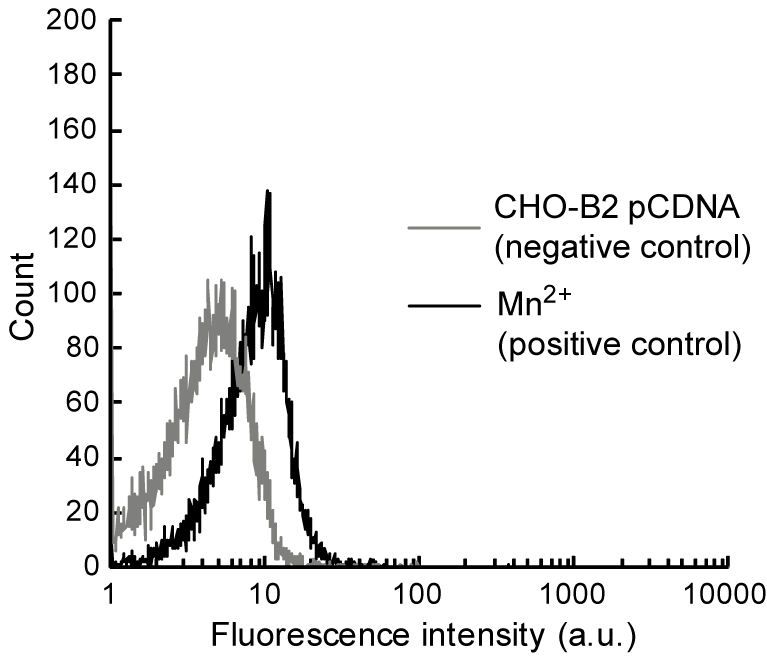

Supplement: Figure S1 — Representative flow cytometry fluorescence intensity histograms illustrating WOW-1 Fab binding for CHO-B2 pCDNA cells, which do not express the integrin β3 subunit, and αvβ3 CHO-B2 cells exposed to Mn2+, which activates integrin αvβ3. (TIF) [file pone.0015746.s001.tif]

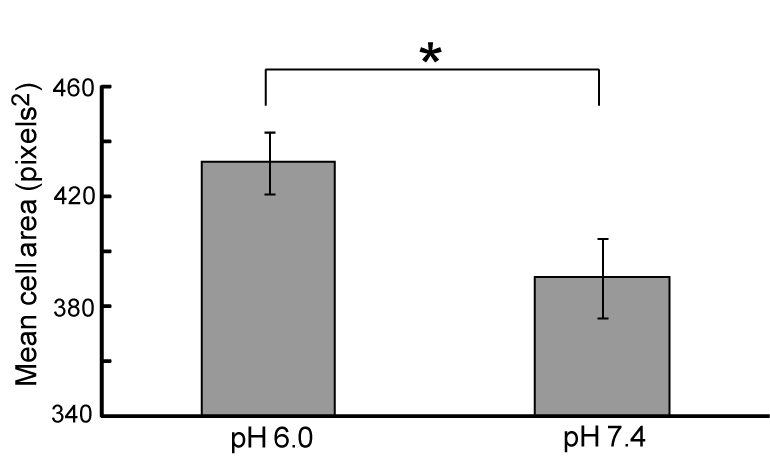

Supplement: Figure S2 — Mean spread area of αvβ3 CHO-B2 cells plated from the suspended state into media at pH 6.0 or pH 7.4. No. 1 glass coverslips or glass-bottom P60 dishes (MatTek) were coated with 15 µg/ml fibronectin in PBS for 1 hour at room temperature. Coverslips or dishes were then rinsed with PBS. αvβ3 CHO-B2 cells were plated on coverslips or dishes in serum-free media with an initial pH of 6.0 or 7.4 and allowed to adhere for 30 minutes in an incubator at 37°C with 5% CO2 before imaging in phase contrast. Media initially set to pH 6.0 remained below pH 7.0 for the duration of the incubation. Cell area was measured using ImageJ. Two independent experiments were performed: in each experiment, approximately 40 cells were measured for each pH. Independent experiments showed consistent results, and data presented are from a single experiment. Results demonstrate that the mean cell area was significantly higher at pH 6.0 than at pH 7.4 (p = 0.0249). Error bars represent SEM. (TIF) [file pone.0015746.s002.tif]

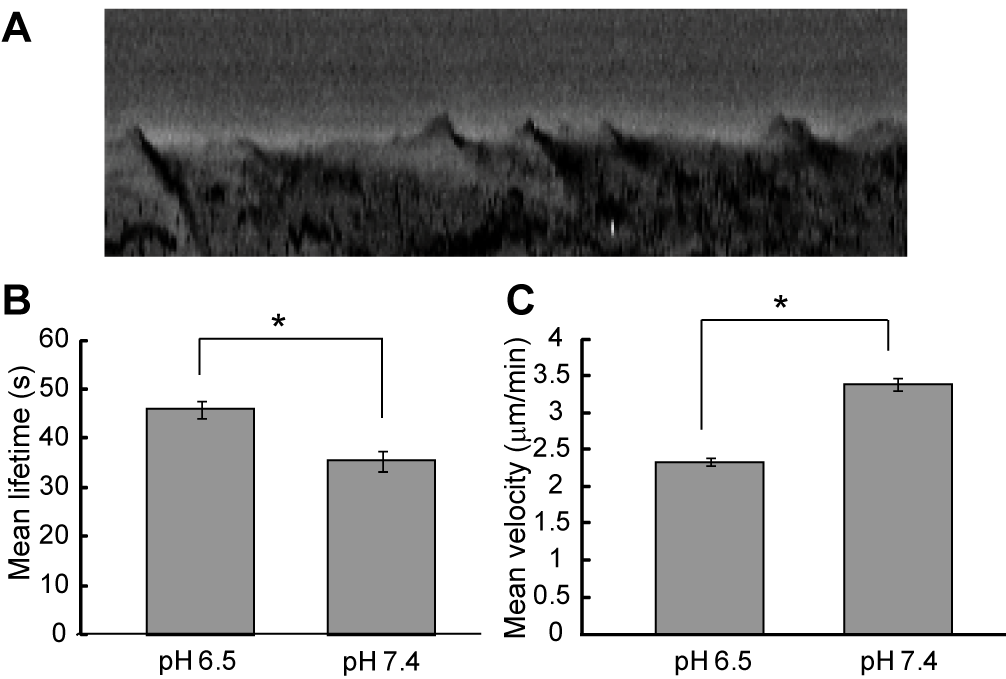

Supplement: Figure S3 — Kymography experiments demonstrate that membrane protrusion lifetime increases and protrusion velocity decreases at acidic extracellular pH. (A) Example kymograph illustrating αvβ3 CHO-B2 membrane dynamics. (B) Mean protrusion lifetime for cells in pH 6.5 or pH 7.4. (C) Mean protrusion velocity for cells in pH 6.5 or pH 7.4. Experiments were conducted as follows: glass-bottom P60 dishes (MatTek) were coated with 30 µg/ml fibronectin in PBS for 1 hour at room temperature. Dishes were then rinsed with PBS. αvβ3 CHO-B2 cells were plated on coverslips or dishes in serum-free media and allowed to adhere for 3 hours before media was changed to bicarbonate-free serum-free media at pH 6.5 or 7.4. Cells were imaged in phase contrast at 40× magnification. Images were collected every 5 seconds for a duration of 25 minutes. Each kymograph was produced by drawing a one-pixel-wide line perpendicular to the cell membrane at an active lamellipod. The images along this line at all timepoints were then sequentially compiled into a single image, illustrating the membrane dynamics at that specific location on the cell. For each visible protrusion event on a kymograph, a straight line was drawn from the beginning of the event to its peak, or to the beginning of a plateau. Events with a height of less than 4 pixels were neglected. The slope of this line represents the protrusion velocity. Protrusion lifetime was quantified as the x-axis projection of this line, with the addition of plateau duration, if applicable. Two independent experiments were conducted: in each experiment, approximately 10–20 cells were imaged and 300–400 protrusion events were analyzed for each pH condition. Independent experiments showed consistent results, and data presented are from a single experiment. Results demonstrate that protrusion velocity is significantly decreased and protrusion lifetime is significantly increased at acidic pH (asterisks represent p<0.001). Error bars represent SEM. (TIF) [file pone.0015746.s003.tif]

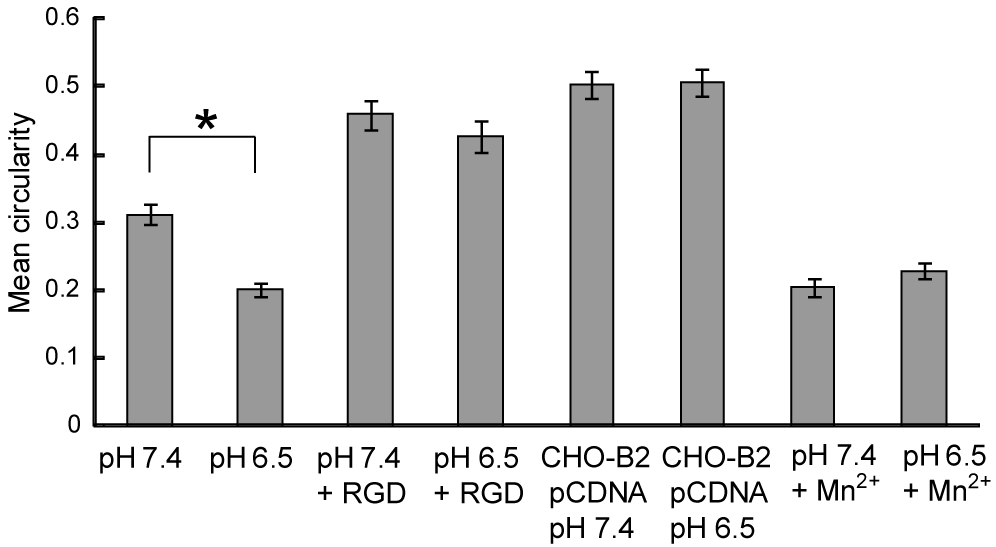

Supplement: Figure S4 — Mean cell circularity 8 hours after changing media pH for adhered αvβ3 CHO-B2 cells or CHO-B2 pCDNA cells. Petri dishes of 50 mm diameter with tight-fitting lids (Pall Life Sciences) were coated with 10 µg/ml fibronectin in PBS for 1 hour at room temperature. Dishes were then rinsed twice with PBS. αvβ3 CHO-B2 or CHO-B2 pCDNA cells were plated on dishes in serum-free media at a density of approximately 6000 cells/cm2 and allowed to adhere for 2 hours before media was changed to bicarbonate-free serum-free media at pH 6.5 or 7.4. MnCl2 (1 mM) or soluble GRGDSPC peptide (200 µg/ml) was also added within this media exchange for some sample conditions, as indicated. Eight hours after the media change, optical images of cells were acquired and analyzed to measure circularity (4πA/P 2, where A is the projected cell area and P is the cell perimeter). At least 50 cells were analyzed for each condition. Results demonstrate that circularity is significantly decreased for αvβ3 CHO-B2 cells in pH 6.5 (Columns 1 and 2, asterisk represents p<0.0001). There was no significant difference in circularity for αvβ3 CHO-B2 cells in the presence of RGD at pH 6.5 vs. pH 7.4 (Columns 3 and 4), CHO-B2 pCDNA cells at pH 6.5 vs. pH 7.4 (Columns 5 and 6), or αvβ3 CHO-B2 cells in the presence of Mn2+ at pH 6.5 vs. pH 7.4 (Columns 7 and 8). (TIF) [file pone.0015746.s004.tif]

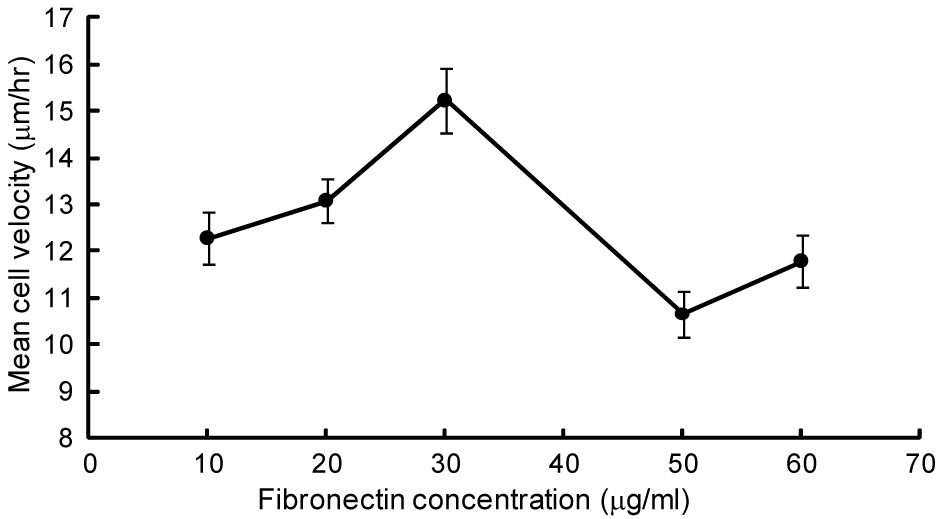

Supplement: Figure S5 — Mean migration speed as a function of fibronectin coating concentration on glass-bottom dishes for cells in media at pH 7.4. Maximum migration speed occurred at 30 µg/ml fibronectin, compared to 1 µg/ml fibronectin when cells are plated on fibronectin-coated polystyrene (Figure 6C, main text). Statistical significance of maximum migration speed value: 10 vs. 30 µg/ml, p<0.01; 30 vs. 50 µg/ml, p<0.001; 30 vs. 60 µg/ml, p<0.001. Error bars represent SEM. (TIF) [file pone.0015746.s005.tif]
